# Body contour adaptation for weight‐loss and bolus for head and neck radiotherapy on Ethos version 2.0 and HyperSight: Synthetic CT versus direct calculation

**DOI:** 10.1002/acm2.14587

**Published:** 2024-12-20

**Authors:** Abby Yashayaeva, R. Lee MacDonald, Amanda Cherpak

**Affiliations:** ^1^ Department of Physics and Atmospheric Sciences Dalhousie University Halifax Canada; ^2^ Department of Radiation Oncology Dalhousie University Halifax Canada; ^3^ Nova Scotia Health Halifax Canada

**Keywords:** adaptive radiotherapy, automated contouring, image‐guided radiotherapy

## Abstract

**Purpose:**

In radiotherapy, body contour inaccuracies may compromise the delineation of adjacent structures and affect calculated dose. Here, we evaluate the un‐editable body contours auto‐generated by Ethos versions 1.0 (v1) and 2.0 (v2) treatment planning softwares for two simulated cases: weight‐loss and bolus application, particularly important for head and neck radiotherapy patients.

**Methods:**

A 3D‐printed target structure was secured to the neck of an anthropomorphic phantom and sequentially covered with silicone boluses of uniform thickness, providing cases for bolus application (0.5 and 1 cm) and weight‐loss (2.0, 1.5, 1.0, 0.5, and 0 cm). HyperSight CBCT images of the phantom were acquired to simulate the online adaptation process. Baseline body contours were manually produced and compared to those auto‐generated in Ethos v1 (synthetic CTs) and Ethos v2 (synthetic CTs and direct calculation on HyperSight CBCTs). Additionally, the target volume D95% dose metric for weight‐loss adapted plans generated by the Ethos v2 were analyzed as a function of surface layer thickness.

**Results:**

The Ethos v1 body contour did not adapt adequately for the weight‐loss image set [mean absolute volume deviation from baseline (MAD) = 205 cm^3^]. The weight‐loss synthetic CT and HyperSight CBCT volumes in Ethos v2 were comparable to manually generated contours (MAD = 34 and 46 cm^3^_,_ respectively); however, the bolus Hypersight CBCT body contour intersected the outer edge of the phantom (MAD = 157 cm^3^). The D95% deviation from the planned dose decreased by up to 10% when using the Ethos v2 adapted plan for the weight‐loss scenario.

**Conclusion:**

Contours in Ethos v1 rely on reference contours and deformable registration algorithms, whereas Ethos v2 does not. Hence, Ethos v2 is preferred for cases involving weight change. A tight‐fitted air gap‐free bolus is critical for achieving accurate body contours for Ethos v2 Hypersight CBCTs.

## 1 INTRODUCTION

The goal of radiotherapy is to ensure sufficient dose coverage to the target while minimizing damage to healthy surrounding tissues. The dynamic approach of adaptive radiotherapy enables adjustments to the patient's plan during the course of treatment to account for changing anatomy.^1^, ^2^ Head and neck (H&N) cancer patients undergoing radiation therapy often experience significant anatomic changes, including weight loss and tumor shrinkage.^1^, ^3^, ^4^ More than half of H&N patients experience a reduction of over 5% in their initial body weight,^5^ which is considered to be critical weight‐loss during radiation therapy.^6^ These changes can alter the dose distribution, originally calculated on the treatment planning fan‐beam computed tomography (CT) image. If substantial volume changes that could compromise outcome occur after treatment begins, patients may require a second planning CT scan and the creation of a new treatment plan.^4^, ^7^, ^8^ This offline re‐planning process may take several days during which patients may either continue receiving treatment based on the original plan, or the treatment could be paused until a new plan is created.^9^

The Varian Ethos system (Varian Medical Systems, Palo Alto, California, USA) is capable of performing online adaptive radiotherapy treatments driven by artificial intelligence (AI) within a 15 to 20‐min time period.^10^, ^11^, ^12^ During an adaptive session on Ethos, a daily kV‐cone beam CT (CBCT) scan is acquired at the unit. Conventionally, direct dose calculations are not performed with CBCT images due to their inferior image quality compared to standard CT images and challenges associated with converting Hounsfield units (HU) to density.^13^, ^14^, ^15^ The standard adaptive Ethos workflow implemented in Ethos version 1.0 (v1) involves creating a synthetic CT (sCT) by registering the planning CT to the CBCT using deformable image registration and mapping the HU of voxels from the planning CT into the CBCT geometry. The software then generates anatomical structures on the sCT by propagating the associated structures from the planning CT to the sCT image using that deformable registration. The physical density data of the sCT is used to perform dose calculations.^16^, ^17^ However, uncertainties in the deformable image registration and discrepancies in the density map between the CT and CBCT may result in an unrealistic sCT and, therefore, an inaccurate dose distribution.^18^, ^19^ For instance, H&N cancer patients often require bolus material to achieve dose at the surface,^20^ and the airgaps that are formed between the surface of the patient and the bolus are difficult to control between treatment sessions. As a result, the density distribution may vary between sessions, leading to inconsistent density information between the CBCT and CT. Wegener et al. demonstrated that discrepancies in air gaps between the planning CT and CBCT led to high variability in bolus thickness in the sCT, with a typical error of 5 mm or more.^21^ A recent publication has demonstrated high dosimetric accuracy of post‐processing CBCT algorithms.^22^ Additionally, the new advanced imaging platform, Hypersight (Varian Medical Systems, Palo Alto, California, USA), has been shown to produce CBCT images with image quality and HU accuracy comparable to standard fan‐beam CT images,^23^ within a 6‐s acquisition time. Together, the Ethos adaptive therapy system paired with the HyperSight imaging platform has the potential to eliminate the need for offline re‐planning, as well as the necessity for a sCT in an online adaptive workflow by using daily HyperSight images for direct dose calculation.

In the next‐generation Ethos version 2.0 (v2), an AI deep‐learning segmentation algorithm is used to automatically generate a specific set of organ structures, including the body structure, directly on the CBCT image without the influence of reference contours. If the image acquired during the adaptive session is identified as a Hypersight CBCT, the voxel values of the CBCT are directly used for dose calculations without the need of a sCT. Otherwise, a sCT is generated in the same manner as for Ethos v1.^24^ For both Ethos versions, the target volumes are propagated from the planning CT to the CBCT using deformable image registration.^12^, ^16^, ^24^ Anatomical structures and target volumes can typically be reviewed and edited during an online adaptive session; however, it is not possible to modify the body structure even though it is a critical variable in dose calculation accuracy. Furthermore, anatomical structures cannot be created outside of the body structure, so any intersections of the body structure with the surface of the patient may compromise the accuracy of other nearby structure contours and their calculated doses. This is particularly important for H&N cancer patients in an adaptive scenario for two reasons: (i) they commonly undergo weight loss during treatment and (ii) have superficial targets that lie in close proximity to the body contour. As there is no ability to modify a body contour during an online adaptive session, understanding the auto‐contouring limitations and assumptions is important information prior to treatment.

A quantitative analysis of the un‐editable body structures in Ethos has not been previously reported for cases of varying volumetric geometry and bolus placement. Here, we assess and compare the accuracy of automatically generated body contours produced by Ethos v1 and the next‐generation Ethos v2 treatment planning software using sCT and HyperSight CBCT images of an anthropomorphic phantom in the H&N region. Additionally, we carry out the first evaluation of adaptive dosimetric properties on Ethos v2 for a planning target volume (PTV) within the same region.

## 2 METHODS AND MATERIALS

### 2.1 Phantom design and data collection

**FIGURE 1:**
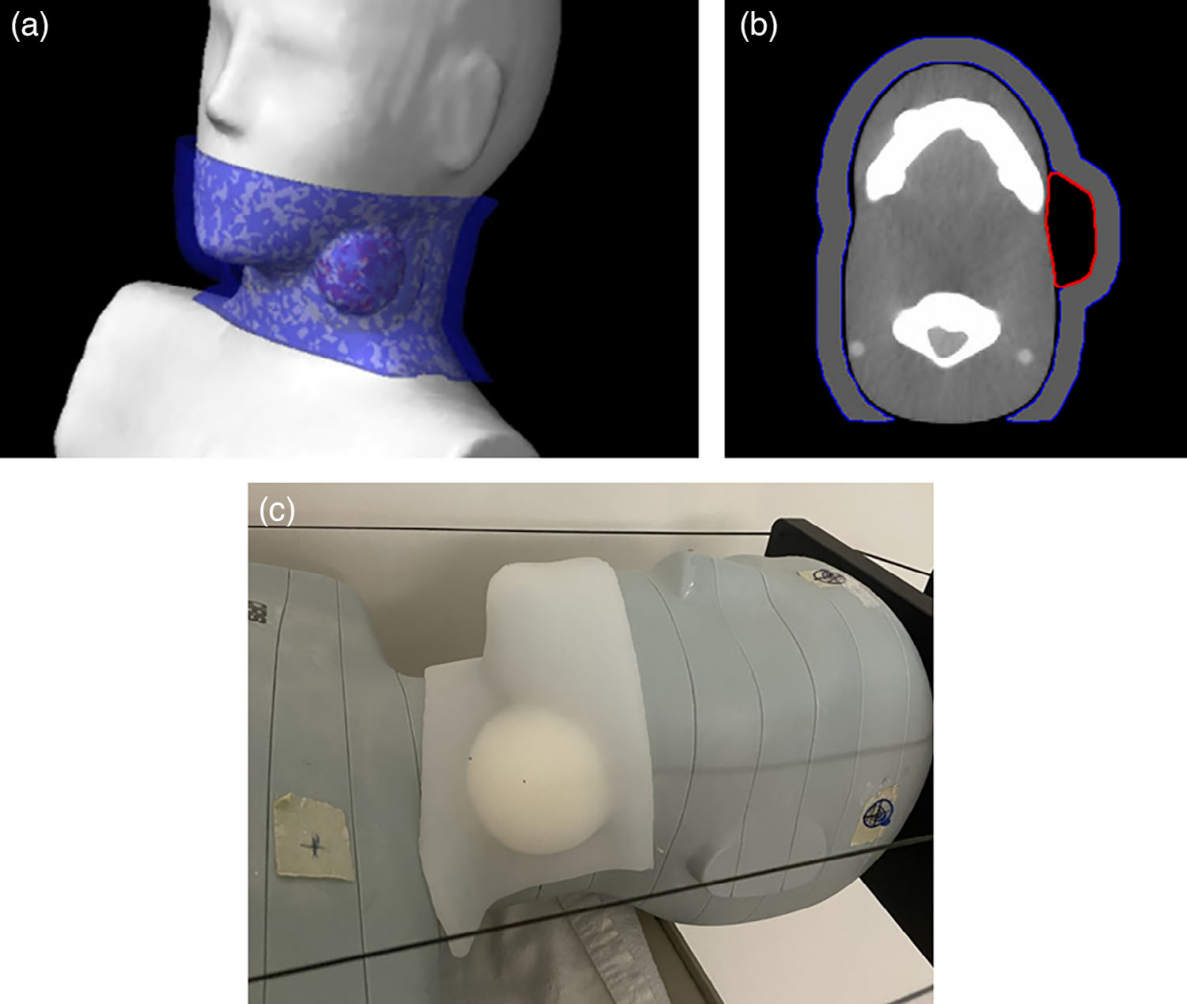
The design of the PTV (outlined in red) and 1.0 cm bolus (outlined in blue) on a CT scan of the anthropomorphic phantom (a), and an axial slice through the structures (b) is shown from the Eclipse treatment planning system. In (c), the PTV is attached to the phantom with the 1.0 cm silicone bolus covering the neck region.

A hemispherical‐shaped PTV structure, measuring 23 cm^3^ in volume, was delineated in the Eclipse treatment planning system (Varian Medical Systems, Palo Alto, California, USA) in the upper jugular anterior nodal region (level IIA node) of an anthropomorphic phantom (ATOM Phantom Family, Adult Female, Sun Nuclear). This region was chosen because level II nodes are susceptible to metastases from cancers originating in the nasal cavity, oral cavity, nasopharynx, oropharynx, hypopharynx, larynx, and the major salivary glands.^25^ Boluses with uniform thickness of 0.5, 1.0, 1.5, and 2.0 cm were created to cover from the phantom's mouth to the clavicle and extended posteriorly. Figure 1a, b show the design of the PTV and 1 cm bolus, as an example, in the treatment planning system. The Digital Imaging and Communications in Medicine (DICOM) format data files of the structures were imported and processed in the Simple Bolus software (Adaptiiv Medical Technologies Inc., Halifax, Nova Scotia, Canada), then sent to Adaptiiv Medical Technologies Inc. for fabrication. The PTV structure was 3D printed from polylactic acid material, while the bolus structures were generated by 3D printing moulds with HP (HP Inc, Palo Alto, California, USA) Multi Jet Fusion and filling them with silicone. The PTV was secured to the phantom's neck using double sided tape, replicating the placement in the treatment planning system. The neck region was sequentially covered with silicone boluses of decreasing thickness, providing two clinical case studies: bolus application (0.5 and 1.0 cm bolus) and weight‐loss (2.0, 1.5, 1.0, 0.5, and 0 cm surface). Figure 1c shows the setup of the phantom on the treatment couch with the PTV attachment covered by the 1.0 cm thick bolus. HyperSight CBCT images of the phantom were acquired using the head preset protocol (100 kV, 88 mAs), and reconstructed with the iCBCT Acuros algorithm.

### 2.2 Data preprocessing

Small air gaps persisted between the bolus and the phantom's outer edge despite careful placement of the target and bolus. For assessment of weight‐loss, the HU values of the bolus and airgaps were adjusted in MATLAB (MathWorks, R2023a) to match that of the phantom and remove the effect of these air gaps. As a first step, any pixels in the image with values less than ‐200 HU were classified as air and flagged temporarily with not‐a‐number (NaN) values, then replaced with their original values in the final step. For each slice, the differences between consecutive pixels in each row were examined to identify spikes in HU at the airgap interface. Differences between adjacent pixels exceeding 45 HU led to poor interpolation over the airgap, so these pixels were also replaced with NaN values to better constrain the interpolation process. The HU values of the bolus were determined to lie within the range of 100–600 HU. Consequently, pixels with values falling within this range were replaced with HU values similar to the background of the phantom. This involved setting the HU of the bolus to a random value with mean and range comparable to that of the phantom background (generated using the “rand” function in MATLAB). For each slice in the image, each row was interpolated to fill the airgaps between the inner edge of the bolus and the surface of the phantom. Lastly, the remaining NaN values outside of the outer edge of the bolus and phantom were replaced with their original values. Real bolus was used to simplify the process of synthesizing the weight loss image set by providing a physically realistic outline of the modified phantom. The steps described above were taken to mitigate the unrealistic HU values caused by the bolus and air gaps, ensuring a uniform background. For the bolus application case, the images set was not modified to assess the effect of the air gaps.

### 2.3 Ethos emulator adaptive workflow

Ethos v1 and v2 emulators, which allow for offline simulation of the commercial adaptive Ethos treatment, were used to replicate the online adaptation process on the acquired images. The HyperSight CBCT of the phantom with the 2.0 cm thick bolus was used for the reference plan generation. This was accomplished by offline editing of DICOM image information. Adaptive plans were created in the Ethos v1 emulator for a sCT workflow, using deformable registration between the reference HyperSight CBCT used for initial planning and the session HyperSight CBCT. The same was done in the Ethos v2 emulator using the sCT workflow. A second workflow was simulated using the Ethos v2 emulator, whereby the session Hypersight CBCT was used for direct dose calculation, eliminating the transfer of density information from the reference image. In all cases, a prescription of 70 Gy in 35 fractions was applied. The PTV dose goals were set to D95% > 70  Gy and D99% > 66.5 Gy. The weight‐loss adaptation process was simulated over five sessions with the phantom surface layer thickness of 2.0, 1.5, 1.0, 0.5, and 0 cm. Separate sessions were simulated for the 0.5  and 1.0 cm bolus for the bolus application case study. For the 0 cm thickness plan, a skin sparing margin was introduced by cropping the PTV back 3 mm from the surface. This structure was used for the optimization to avoid enforcing coverage of the target at the surface. The scheduled (reference plan on daily anatomy) and adapted PTV dose properties were recorded, and the auto‐segmented structures, scheduled plans, and adapted plans were exported from the Ethos emulator treatment management system, then imported into the Eclipse treatment planning system for further evaluation.

### 2.4 Body structure quantification

The body contours were cropped in the superior/inferior direction for all cases (*Z* = −4.9 cm to *Z* = 2.5 cm) in the Eclipse treatment planning system to include only the region where the bolus was present to allow for consistent comparison, and the volumes of the structures were recorded. For each session image, manual contours of the body structures were reproduced three times using a threshold of −300, −450, and −600 HU, and the average volume was used as a baseline, with the standard deviation reflecting the baseline uncertainty. Volume differences of the body structures generated in Ethos v1 (sCTs) and Ethos v2 (sCT and HyperSight) were calculated relative to the baseline structures, and the mean absolute volume deviation (MAD) from the baseline was assessed across the five weight‐loss sessions. In a clinical setting, the type of bolus used during treatment typically does not change between sessions so the body structures generated from the deformation onto a sCT remains stable, even with varying airgaps.^21^ However, the Ethos v2 Hypersight workflow auto generates the body structure from scratch for each session using an AI deep‐learning segmentation algorithm, so it was of particular interest to review those contours. The volume analysis described above was repeated for the two bolus cases from the Ethos v2 Hypersight adaptive sessions.

The coefficient of determination (*R* ^2^%) was used to assess the relationship between the body structure volume and the surface layer thickness for each weight‐loss adaptive case, as well as for the baseline structures, defined in Equation 1. The variance is defined in Equation 2 where *N* is the number of data points, and μ is the average of all $x_{i}$ data points.

(1) $$R^{2}\%\;=100\%-\;\frac{var\left(y\;-y_{fitted}\right)}{var\left(y\right)}\;*100\%$$

(2) $$var\;\left(x\right)=\frac{1}{N}\;\sum_{i=1}^{N}{\left(x_{i}-\mu \right)}^{2}$$

### 2.5 Dosimetric evaluation

The PTV D95% dose metric for the five adapted plans generated by the Ethos v2 emulator with HyperSight was analyzed as a function of surface layer thickness. The adapted plans were forward calculated for the clinical Ethos machine in the Eclipse treatment planning software using the same dose grid resolution (0.25 cm) and dose calculation algorithm (Acuros External Beam) as in the emulator software. A gamma evaluation was performed using the VeriSoft software (PTW, Freiburg, Germany) to compare the emulator dose data to the forward calculated dose and confirm consistency with the clinical setting. The gamma metric quantifies the agreement between the two dose distributions by considering both dose difference (DD) and distance‐to‐agreement (DTA) criteria.^26^ The percentage passing rate indicates the proportion of points in the dose grid that meet the acceptance criteria. For photon beams, a 90% passing rate with the current clinical standard of a 3% DD criterion and a 3 mm DTA criterion is recommended.^27^

## 3 RESULTS

**FIGURE 2:**
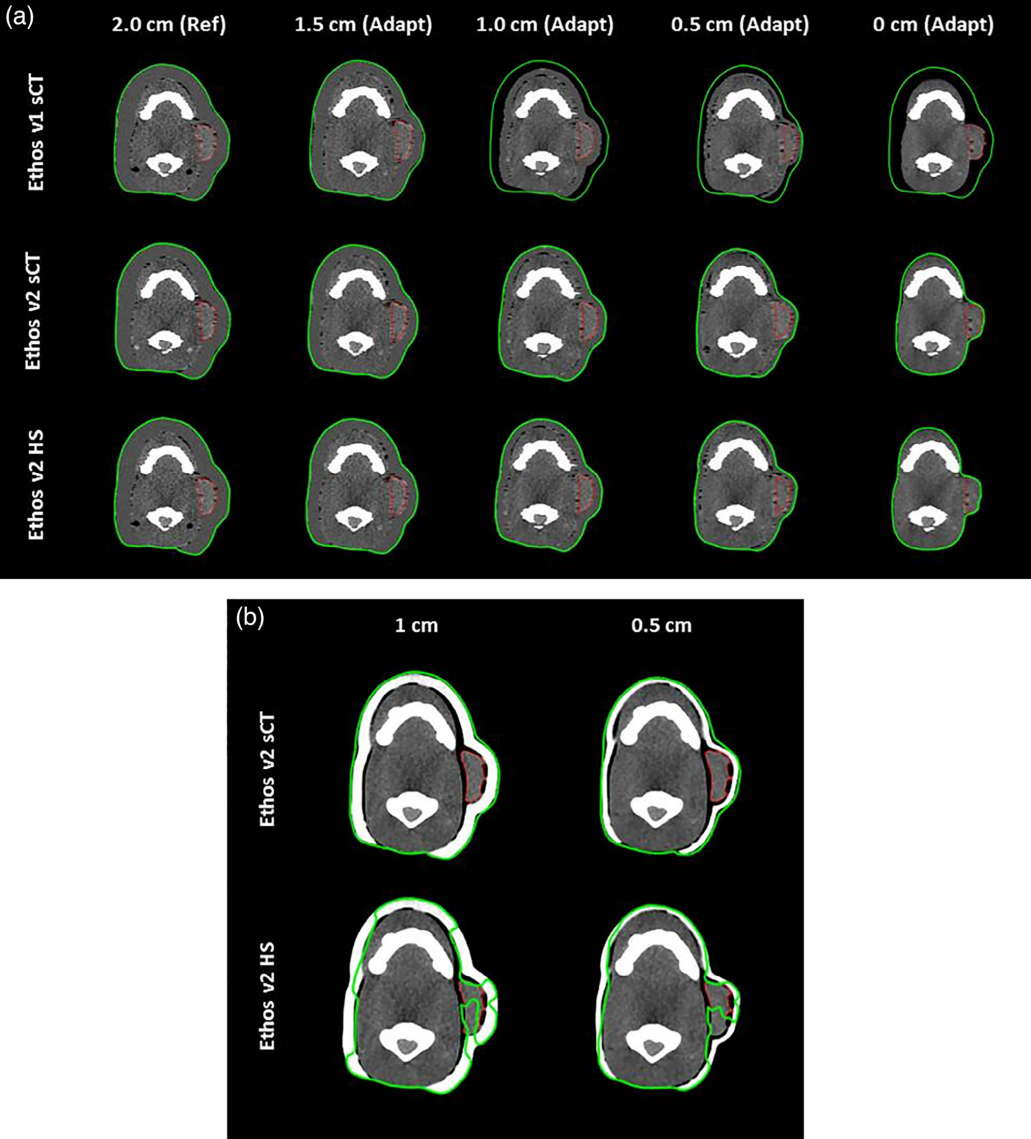
(a) The Ethos‐generated body contours in green, adapted to decreasing surface layer thickness. The reference plan was created on the phantom image with a 2.0 cm thick surface layer, while the others were used for adaptive sessions. (b) The Ethos‐generated body contours for the phantom with a 1.0 cm bolus (left) and 0.5 cm bolus (right).

The adapted body contours are displayed in Figure 2a for the phantom with decreasing surface layer thickness (left to right) for Ethos v1 with sCT (top row), Ethos v2 with sCT (middle row), and Ethos v2 with Hypersight (bottom row). In Ethos v1, the contour failed to conform to the surface of the phantom for simulated weight‐loss. The body contours for the two bolus cases are shown in Figure 2b for Ethos v2 with sCT (top row) and Ethos v2 with Hypersight (bottom row). The AI auto‐segmentation algorithm of the Ethos v2 Hypersight workflow resulted in the body contour intersecting the bolus, consequently producing an inaccurate contour of the adjacent PTV. This was generally the case for airgaps larger than 2.5 mm; however, the body contour also exhibited unpredictable behavior for smaller airgaps (< 0.4 mm).

**FIGURE 3:**
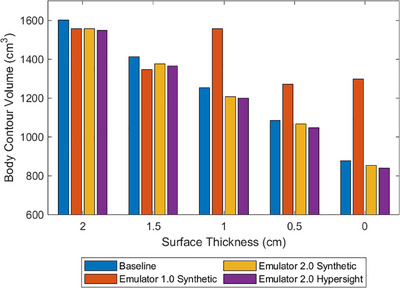
The volumes of Ethos‐adapted body contours for weight‐loss simulated images. The baseline contour was generated in the Eclipse treatment planning system.

The body contour volumes for all surface layer thicknesses and comparison with baseline volumes are presented in Figure 3. The maximum standard deviation of baseline body contour volumes obtained from the three thresholding methods, across all session images, was 53 cm^3^, serving as the measure of uncertainty. The Ethos v2 sCT and HyperSight mean absolute volume differences were within uncertainty of the baseline volumes (MAD = 34 and 46 cm^3^, respectively); however, this was not the case for Ethos v1 (MAD = 205 cm^3^). The contour volumes of the phantom with both 0.5  and 1.0 cm clinical bolus application agreed with the baseline volume within uncertainty for Ethos v2 with sCT (MAD = 10 cm^3^); however, the contour intersected the phantom for both bolus cases with Hypersight (MAD = 157 cm^3^). The relationship between the body structure volume and the surface layer thickness is evident in Figure 3. A strong linear trend is present for both the sCT and Hypersight workflows in Ethos v2 (*R* ^2^ = 99.6% and 99.8%, respectively), whereas Ethos v1 exhibits a comparatively weaker relationship (*R* ^2 ^= 44.7%).

**FIGURE 4:**
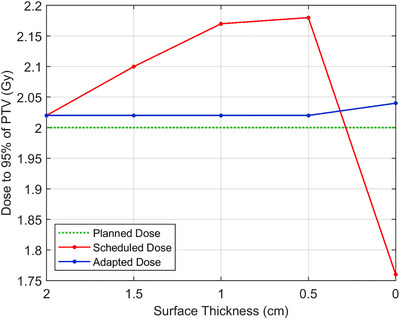
The PTV D95% with decreasing surface thickness for an Ethos v2 emulator adaptive session with Hypersight, simulating weightloss. The planned dose reflects the dose achieved by the reference plan on the original planning CT. The scheduled dose is obtained from calculating the reference plan on the session image, and the adapted dose is the new plan generated during the Ethos online session, calculated on the session image.

The PTV D95% dose metric for the five adapted plans generated by the Ethos v2 emulator with HyperSight are plotted as a function of surface layer thickness in Figure 4. The dose per fraction delivered from the scheduled plan varied by up to 12% of the plan dose with decreasing surface thickness, while the adapted dose remained within 2% of the planned dose. Gamma 3%/3  mm pass rates from the comparison of the adapted plan dose data from the emulator and the same plan forward calculated in the Eclipse treatment planning software were 99.9%, 98.3%, 98.4%, 100%, 99.4% for a surface thickness of 0, 0.5, 1.0, 1.5, and 2.0 cm, respectively. Both Ethos V2 sCT and HS workflows showed comparable dose adaptation, with differences in PTV D95% of less than 0.02 Gy for the adapted plans. Differences in PTV D95% of less than 0.13 Gy were observed between the workflows for the scheduled plans.

## 4 DISCUSSION

The correct delineation of organ and target volumes is a critical factor in radiation therapy to ensure dose calculation accuracy. The Varian Ethos system aims to perform online adaptive radiotherapy treatments driven by AI. However, in the adaptive workflow, it is not possible to edit the body contour, which could also potentially compromise the accuracy of adjacent structure contours and resultant dose estimations. It is paramount to understand the performance of the body contour generation, as the adaptive workflow takes place under tight time constraints with a patient on the treatment couch and edits are not possible. Anecdotal reports from groups treating H&N have included the need to cut out sections of the mask or repeat FBFCT simulation when problems with the body contour during the adaptive process are encountered. This work provides the first comparative evaluation of the next generation of the Ethos platform, version 2.0, along with state‐of‐the‐art HyperSight CBCT, and aims to characterize the performance of the system in two clinically relevant scenarios. Boluses of varying thickness were used to cover the neck of an anthropomorphic phantom, simulating weight‐loss during H&N radiation treatment, as well as the clinical use of a bolus. Both weight‐loss and bolus treatment cases had boluses of uniform thickness involved in the experimental design. However, for testing the effects of the boluses, no preparatory image modification was required, while for simulating weight loss, both airgaps and bolus were identified in the images and adjusted to match the HU of the phantom.

To evaluate the adaptive performance for weight‐loss, a Hypersight CBCT of the phantom with the 2.0 cm thick surface layer was employed for generating a reference plan, while the successive CBCTs with decreasing surface thickness were used for the adaptive sessions in the emulator platform. When a geometrical change is introduced in Ethos v1, the deformation of the planning CT may not perfectly match the session CBCT, which could potentially result in a sCT that is not representative of anatomy during treatment. When decreasing the bolus thickness, the contour volume versus surface thickness will deviate from the expected linear relationship if the contours are generated incorrectly. *R* ^2^ indicates how well a linear fit describes the data, and therefore the accuracy of the contour. Ethos v1 exhibited an overestimation of the body structure volume during the weight‐loss adaptive sessions (MAD = 205 cm^3^, *R* ^2 ^= 44.7%). On average, the volumes of adapted contours from Ethos v2 sCT and HyperSight workflows were within the uncertainty range of the manually generated baseline volumes (< 53 cm^3^), demonstrating a strong linear trend correlating with the decrease in surface thickness (*R* ^2^ > 99%). A body contour that closely resembles one drawn manually can alleviate concerns about the accuracy of the predicted dose, ensuring it physical reflects the real anatomy. The notable improvement in contouring performance of Ethos v2 for weight‐loss prompted further assessment of the adapted plan. After confirming dose consistency between the plans calculated in the emulator versus the clinical treatment planning software, with all plans retaining a high gamma pass rate (> 98%), the dosimetric properties from the Ethos v2 adaptive plans with Hypersight were analyzed with respect to surface thickness. The variation in D95% between the planned dose and the scheduled plan calculated on the session images decreased by up to 10% when implementing the adaptive plan. The deviations of Ethos v2 HyperSight contours from the manually generated contours were within the uncertainty (the maximum standard deviation of baseline body contour volumes obtained from different thresholding methods, across all session images), so we can assume that the dose calculation in this case was not affected by discrepancies of the body contour. The differences in PTV D95% between HyperSight and sCT workflows were likely due to HU inaccuracies in the sCT.

Given that the Ethos v2 Hypersight workflow does not rely on prior information from the planning CT, but rather generates contours for each session independently using a deep‐learning segmentation algorithm, the secondary objective was to assess how effectively Ethos v2 generates contours on a Hypersight session image when a bolus is present. The bolus was fitted to the phantom to minimize the air gaps; however, similar to clinical scenarios, it was challenging to eliminate airgaps between the skin surface and bolus entirely. The contour volumes of the phantom with both 0.5 and 1.0 cm clinical bolus application agreed with the baseline volume within uncertainty for Ethos v2 with sCT (MAD = 10 cm^3^); however, the contour intersected the phantom for both bolus cases with Hypersight (MAD = 157 cm^3^). Furthermore, the body contour intersected the PTV, which cannot be contoured outside of the body. Generally, the auto‐contouring failed for airgaps larger than 2.5 mm. Even if it was possible to achieve airgaps of less 2.5 mm throughout the entire bolus, there were still cases where the body contour exhibited unpredictable behavior. This limitation could be resolved if the user had the option of editing the body contour. The dose is only evaluated within contours, so the under‐segmentation of the body and PTV would result in a plan that may not deliver sufficient dose to the target volume, or inaccurately estimate dose to healthy tissues. This could pose a challenge in a clinical environment since there is no option to adjust the body contour during the online session. The poor performance of AI contours for the bolus scenario compared to weight‐loss is likely due to the airgaps between the bolus and the skin. The algorithm first considers all regions with a density greater than −200 HU as the possible outline when localizing the body. Regions that are not fully connected and are located at a distance outside of a tolerated range from the image center are then rejected from the contour.^16^ To further refine the body outline, the auto‐segmentation library in Ethos v2 attempts to recognize external devices such as masks, wires, molds, etc., and exclude them from the contour. The bolus might be recognized as an external device, resulting in its exclusion from the body contour.^28^

As Ethos continues to emerge in radiation therapy, we anticipate that our study will provide valuable insights for the field of adaptive radiotherapy. The importance of an airgap‐free bolus has been previously presented in the context of synthetic CT accuracy on Ethos v1.^21^ Ethos v2 employs a deep learning algorithm to generate contours instead of relying on deformable image registration, thereby eliminating the need for synthetic CT. This study addresses the accuracy of body contour generation and involves a comparison between different versions of the software. Additionally, Ethos's adaptation to weight loss has not been documented in prior research. One of the aims of this study is to raise clinical awareness of the software limitations before a patient is present on the bed. A clinician may decide whether to refer the patient for an alternative treatment approach, such as using a non‐adaptive treatment machine if bolus application is required. This work may also help Ethos users weigh the trade‐offs between the two versions when preparing for an upgrade, such as determining whether improved body contour performance is more crucial for weight loss (Ethos V2.0) or bolus (Ethos V1.0 and V2.0 with sCT). Additionally, it may inform the commissioning tests necessary during the upgrade process.

This study focused solely on the H&N region; however, it is reasonable to expect similar behavior to be observed in other sites. Specifically, changes in geometry may lead to discrepancies between the sCT and the CBCT in Ethos v1, while introduction of new structures or densities outside of the body may not be accounted for in the deep‐learning segmentation algorithm in Ethos v2. There are important challenges that can be investigated using phantoms that cannot be addressed with patient data such as performing repeated scans, incrementally increasing bolus thickness, and controlling airgaps. Our phantoms design allowed us to accentuate and isolate the behavior of the system concerning particular variables. We were able to simulate cases that might not be fully represented from a patient cohort, such as more extreme weight‐loss, and to push the system to evaluate its limits. The performance observed under these conditions suggest that Ethos 2.0 is likely to function effectively in typical clinical scenarios.

However, to confirm this, we have initiated a follow‐up investigation involving real patient data where certain cases from the cohort can be selected based on realistic weight‐loss achieved during treatment. This forthcoming study will focus on H&N patient imaging on the Ethos machine, with images acquired at a minimum of two time‐points during treatment. Preliminary results from this patient cohort have demonstrated similar adaptations in body contours and bolus exclusion, consistent with the results of our present phantom study. A detailed analysis of the patient data will be presented in future publications. Incorporating real patient data and exploring additional factors, such as the dosimetric evaluation of realistic target volumes, will be important for translating our findings into practical applications within a clinical setting. Furthermore, a more complex design of the experiment would include the evaluation of contours of surrounding organs and their respective doses.

## 5 CONCLUSION

Adjusting the thickness of the phantom surface relative to the planning CT during the adaptive treatment sessions in Ethos v1 led to inadequate body contour adaptation, indicating influence from the planning CT and reference contours. Ethos v2 demonstrated superior performance compared to v1, with body structure volumes falling within the uncertainty range of those manually generated in the treatment planning software. This makes Ethos v2 the preferred choice for cases involving weight change. On the contrary, the AI auto‐segmentation algorithm failed to recognize the bolus as part of the body contour, so a tight‐fitted air gap‐free bolus is critical for achieving accurate body contours. These findings are crucial for understanding how to navigate the constraints of scenarios within the new platform; however, further work is needed validate these results in a realistic case involving weight‐loss and the application bolus.

## AUTHOR CONTRIBUTIONS

Abby Yashayaeva, Lee MacDonald, and Amanda Cherpak contributed to the design of the work, data acquisition, analysis and interpretation of data. Abby Yashayaeva drafted the manuscript, and Lee MacDonald and Amanda Cherpak critically reviewed and revised it. All authors approved the final version to be published and agree to be accountable for all aspects of the work in ensuring that questions related to the accuracy or integrity of any part of the work are appropriately investigated and resolved.

## CONFLICT OF INTEREST STATEMENT

Abby Yashayaeva has no conflicts of interest to declare. Amanda Cherpak and Lee MacDonald are members of an advisory group with Varian.

## Data Availability

The data that support the findings of this study are available from the corresponding author upon reasonable request.
